# Association between anesthetics and the postoperative pneumonia risk in patients with non-traumatic subarachnoid hemorrhage: an analysis of the MIMIC-IV database

**DOI:** 10.3389/fneur.2025.1615897

**Published:** 2026-01-08

**Authors:** Ying Wang, Fangling He, Fu Guo

**Affiliations:** 1 Department of Anesthesiology, Shaoxing Xinchang County People’s Hospital, Shaoxing, Zhejiang, China; 2 Department of Anesthesiology, Zhuji Central Hospital, Zhuji, Zhejiang, China; 3 Department of Anesthesiology, Shaoxing Shengzhou Maternal and Child Health Hospital, Shaoxing, Zhejiang, China

**Keywords:** anesthetic, fentanyl, postoperative pneumonia, non-traumatic subarachnoid hemorrhage, MIMIC

## Abstract

**Objective:**

Postoperative pneumonia (POP) is a common complication in surgical patients. The choice of anesthetic may affect POP in patients with non-traumatic subarachnoid hemorrhage (SAH). This study aims to identify a key anesthetic as an independent predictor for POP risk in patients with non-traumatic SAH.

**Methods:**

This retrospective study utilized data from the MIMIC-IV database spanning the period from 2008 to 2019. First, receiver operating characteristic (ROC) curve analysis, decision curve analysis, and factor importance analysis were conducted to determine which anesthetic was more effective and important in predicting POP in patients with non-traumatic SAH. Second, three different multivariate logistic regression models were established to investigate the association between fentanyl use and the risk of POP, followed by subgroup analysis. Finally, a series of comparative analyses were conducted between fentanyl and traditional disease severity scores.

**Results:**

Fentanyl (AUC: 0.680) demonstrated a significantly higher predictive value than propofol (AUC: 0.604), midazolam (AUC: 0.608), and dexmedetomidine (AUC: 0.630) in predicting POP in patients with non-traumatic SAH (all Delong test *p* < 0.05). Multivariate logistic regression analysis revealed that fentanyl remained significantly associated with POP after adjustment for various confounders (Model 1: OR = 4.979, 95%CI: 3.652–6.874; Model 2: OR = 2.965, 95%CI: 2.138–4.152; Model 3: OR = 4.433, 95%CI: 3.239–4.152). CHF and CVD significantly modified the association of fentanyl with POP. Further, fentanyl demonstrated satisfactory clinical value and increased the predictive efficacy of the traditional disease severity scores.

**Conclusion:**

Our findings indicated that fentanyl was associated with POP and may serve as a robust predictor of POP risk in patients with non-traumatic SAH.

## 1 Introduction

Non-traumatic subarachnoid hemorrhage (SAH) is a life-threatening type of hemorrhagic stroke primarily caused by intracranial aneurysm rupture. With an incidence of ~9 per 100,000 people annually, non-traumatic SAH constitutes 2–7% of all strokes and leads to high risk of disability and mortality (1–5). The majority of non-traumatic SAH patients admitted to the intensive care unit (ICU) are at a high risk of infection and will suffer from postoperative complications even after advanced treatment such as endovascular coil embolization (6). As a common complication, postoperative pneumonia (POP) significantly impacts the prognosis of non-traumatic SAH patients, with a reported incidence rate of 27.2% (7). Studies demonstrated that ICU patients with POP have a threefold higher mortality rate, longer hospital stays, and significant healthcare and socioeconomic burdens (8–11). Therefore, identifying a marker for timely and accurate prediction of POP can optimize risk assessment and benefit risk stratification in ICU-admitted non-traumatic SAH patients.

Pain management constitutes the core of ICU management, so the selection of anesthetics has attracted significant attention. The choice of anesthetics may influence the risk of POP by cardiopulmonary function, gastrointestinal activity, immune regulation and inflammatory response (12). Fentanyl, propofol and midazolam have immunosuppressive properties, while dexmedetomidine does not (13). Additionally, fentanyl shows pro-inflammatory effects, while the other drugs have anti-inflammatory effects (14, 15). However, an animal study showed that dexmedetomidine failed to improve pneumonia related to stroke in mice (16). Nevertheless, there is a lack of research to examine which anesthetic can be more helpful in predicting POP in patients with non-traumatic SAH.

Hence, this study aimed to identify a key anesthetic drug as an independent predictor of POP risk in patients with non-traumatic SAH. We hypothesize that fentanyl may be this key anesthetic drug. The significance of this study lies in optimizing anesthetic choices, enhancing the quality of clinical decision-making, and further improving risk stratification and predictive models.

## 2 Methods

### 2.1 Data source and study participants

This study employed a retrospective observational design, utilizing data from the Medical Information Mart for Intensive Care (MIMIC-IV) database (version 2.2). MIMIC-IV is a publicly available medical database jointly developed by the Massachusetts Institute of Technology (MIT) and Beth Israel Deaconess Medical Center. It contains anonymous clinical data of over 50,000 patients in the ICU from 2008 to 2019. The database includes structured and unstructured data such as vital signs, laboratory tests, medication records, imaging reports, nursing notes, and disease diagnoses (such as ICD codes), covering various ages, diseases, and treatment scenarios. To safeguard patient privacy, all personal information was de-identified by replacing it with random codes. As a result, neither patient consent nor ethical approval was required for this study. The research team has obtained authorization to use the database and extract data. This study was conducted and reported in accordance with the STROBE guidelines.

**Figure 1:**
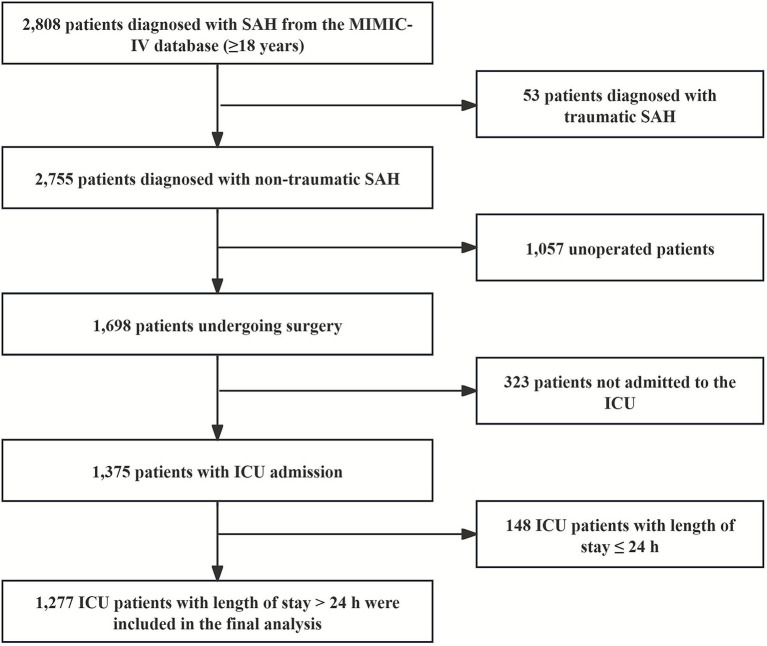
Flowchart of participant selection.

We recruited 2,808 cases of SAH, aged 18 years or above, who had their first documented ICU admission between 2008 and 2019, based on ICD-9 code 430 and ICD-10 codes I60 to I609 (2). The exclusion criteria were as follows: (1) individuals diagnosed with traumatic SAH; (2) individuals who have not undergone surgery; (3) patients who were not admitted to the ICU or whose duration of stay in the ICU was ≤1 day (17). After applying these criteria, a total of 1,277 patients were identified for analysis (Figure 1).

### 2.2 Exposure and clinical outcome

The primary exposure variables of interest were the four types of anesthetics, including propofol, midazolam, dexmedetomidine, and fentanyl. The primary endpoint was the risk of POP in non-traumatic SAH patients. POP was diagnosed according to the ICD codes. Study participants were divided into the non-POP group (*n* = 955) and the POP group (*n* = 272).

### 2.3 Data collection

In addition to the exposure variables and clinical outcome, this study also extracted data from five major domains: (1) Demographic data, including age, gender, and marital status. (2) Physiological indicators, including heart rate, systolic blood pressure (SBP), diastolic blood pressure (DBP), and percutaneous oxygen saturation (SpO_2_). (3) Preexisting comorbidities, such as hyperlipidemia, diabetes, congestive heart failure (CHF), cerebrovascular disease (CVD), chronic pulmonary disease, and renal disease. (4) Clinical severity indicators, including Simplified Acute Physiology Score II (SAPSII), Glasgow Coma Scale (GCS), Acute Physiology Score III (APSIII), and World Federation of Neurosurgical Societies (WFNS) grade. (5) Treatment interventions, including endovascular intervention and mechanical ventilation. (6) Laboratory indicators, including red blood cell distribution width (RDW), hemoglobin, glucose, white blood cell (WBC), platelets, creatinine, anion gap (AG), international normalized ratio (INR), prothrombin time (PT) and partial thromboplastin time (PTT).

### 2.4 Statistical analysis

Continuous and categorical variables were expressed as the median along with interquartile range (IQR) and counts with percentages. Differences between groups were evaluated using the t-test or Mann–Whitney U-test and Chi-square test or Fisher’s exact test. To address missing data, variables with a missing value proportion of 20% or higher were excluded from the analysis. For variables with a missing value proportion below 20%, missing data were imputed using the Random Forest algorithm. Subsequently, we compared the intergroup differences of the variables after imputation to evaluate the stability of the variables.

Receiver operating characteristic (ROC) curve analysis, decision curve analysis (DCA), and factor importance analysis were conducted to select an efficient and key anesthetic for predicting POP in patients with non-traumatic SAH. Delong test was used to examine the significant differences between the area under curve (AUC).

A multicollinearity assessment was performed, and variables with a variance inflation factor (VIF) exceeding 5 were excluded. Logistic regression examines the relationship between one or several independent variables and a binary dependent variable (18). Therefore, three distinct multivariate logistic regression models were established to investigate the association between the key anesthetic and the risk of POP. Model 1 was adjusted for demographic characteristics and comorbidities including age, gender, CHF, and chronic pulmonary disease; Model 2 was adjusted for vital signs, mechanical ventilation, and scoring systems including heart rate, mechanical ventilation, SAPSII, APSIII, and GCS; Model 3 adjusted for laboratory indicators including RDW, hemoglobin, glucose, WBC and SpO_2_. E-value was proposed to assess potential unmeasured or residual confounding in observational studies and its calculation formula is as follows: OR (odds ratio) = OR + sqrt {OR × (OR − 1)} (19). Hence, we evaluated the robustness by calculating the E-values of the ORs for the association between the key anesthetic and the risk of POP. Subgroup analyses were also conducted for key anesthetic and the risk of POP based on gender (female/male), mechanical ventilation (yes/no), surgical approach (endovascular intervention for aneurysm/others), hyperlipidemia (yes/no), CHF (yes/no), CVD (yes/no), chronic pulmonary disease (yes/no), and renal disease (yes/no). The false discovery rate (FDR) correction for multiple comparison was performed. The interactions between key anesthetic and variables used for stratification were tested using a *Z*-test (20). To clarify the differences in the efficacy of key anesthetic and commonly used clinical disease severity scoring systems in predicting POP, we compared the predictive performance of key anesthetic with that of GCS, SAPSII, and APSIII. The integrated discrimination improvement (IDI) is a statistic proposed as a measure of the incremental prognostic impact that a new biomarker will have when added to an existing prediction model for a binary outcome. Therefore, on the basis of the traditional scoring system, the key anesthetic was incorporated to build a new model, and the efficacy changes were evaluated by calculating the IDI index. All statistical analyses were performed using SPSS version 26.0 and R version 4.2.1. ROC, DCA, and IDI analyses were performed using the R packages “pROC,” “rmda,” and “PredictABEL,” respectively. Results with two-sided *p* values less than 0.05 were considered statistically significant.

## 3 Results

### 3.1 Descriptive results

Herein, a total of 1,277 patients with non-traumatic SAH were enrolled, with a median age of 61 years (IQR: 50–73), with males accounting for 50.28% of the study population (*n* = 617). Patients with POP were older, more likely to be male, had higher heart rates and SpO_2_ levels, more likely to have CHF and chronic pulmonary disease (all *p* < 0.05) (Table 1). Patients with POP exhibited higher SAPSII score, APSIII score, and WFNS grade, had a lower GCS score, and were more likely to use mechanical ventilation (all *p* < 0.05) (Table 1). Moreover, patients who receive propofol, midazolam, dexmedetomidine, and fentanyl are more prone to POP (all *p* < 0.05) (Table 1). The remaining variables, including marital status, hyperlipidemia, diabetes, CVD, renal disease, and surgical approach, showed no significant differences between the two groups (all *p* > 0.05) (Table 1). Higher levels of RDW, hemoglobin, glucose, and WBC count were observed in POP patients, which persisted before and after imputation (all *p* < 0.05) (Tables 2, 3). There was no statistically significant difference in platelets, creatinine, AG, INR, PT, PTT, SBP, and DBP between the two groups, either before or after imputation (all *p* > 0.05) (Tables 2, 3).

**Table 1:** Comparison of demographic and clinical data in patients with non-traumatic SAH according to incidence of POP.

Variable	Overall (*n* = 1,227)	Non-POP (*n* = 955)	POP (*n* = 272)	*p*-value
Demographics				
Age, years	61.00 (50.00, 73.00)	60.00 (50.00, 73.00)	63.00 (52.00, 74.00)	0.045
Gender, *n* (%)				0.026
Female	610 (49.72)	491 (51.41)	119 (43.75)	
Male	617 (50.28)	464 (48.59)	153 (56.25)	
Marital status, *n* (%)				0.091
Married	520 (42.38)	420 (44.00)	100 (36.77)	
Other	707 (57.62)	535 (56.00)	172 (63.23)	
Vital signs				
Heart rate, beats/min	64.00 (57.00, 74.00)	63.00 (56.00, 72.00)	67.00 (59.00, 78.00)	<0.001
SpO_2_, %	85.00 (82.00, 85.00)	85.00 (85.00, 85.00)	85.00 (76.00, 85.00)	<0.001
Comorbidities, *n* (%)				
Hyperlipidemia				0.856
Yes	342 (27.87)	265 (27.75)	77 (28.31)	
Diabetes, *n* (%)				0.053
Yes	226 (18.42)	165 (17.28)	61 (22.43)	
CHF				0.014
Yes	127 (10.35)	88 (9.22)	39 (14.34)	
CVD				0.456
Yes	857 (69.84)	672 (70.37)	185 (68.01)	
Chronic pulmonary disease				0.002
Yes	168 (13.69)	115 (12.04)	53 (19.49)	
Renal disease				0.449
Yes	112 (9.13)	84 (8.80)	28 (10.29)	
Scores				
SAPSII	32.00 (24.00, 40.00)	30.00 (23.00, 39.00)	36.00 (28.00, 45.00)	<0.001
GCS	12.00 (7.00, 14.00)	13.00 (9.00, 14.00)	8.00 (5.00, 11.00)	<0.001
APSIII	34.00 (25.00, 47.00)	32.00 (25.00, 44.00)	42.00 (29.00, 57.00)	<0.001
WFNS Grade, *n* (%)				<0.001
I, II and III	601 (48.98)	545 (57.07)	56 (20.59)	
IV and V	626 (51.02)	410 (42.93)	216 (79.41)	
Treatment, *n* (%)				
Surgical approach				0.099
Endovascular intervention for aneurysm	191 (15.57)	147 (15.39)	44 (16.18)	
Others	1,036 (84.43)	808 (84.61)	228 (83.82)	
Mechanical ventilation				<0.001
No	193 (15.73)	188 (19.69)	5 (1.84)	
Yes	1,034 (84.27)	767 (80.31)	267 (98.16)	
Anesthetic drugs use, *n* (%)				
Propofol				<0.001
Yes	289 (23.55)	181 (18.95)	108 (39.71)	
Midazolam				<0.001
Yes	235 (19.15)	137 (14.35)	98 (36.03)	
Dexmedetomidine				<0.001
Yes	239 (19.48)	131 (13.72)	108 (39.71)	
Fentanyl				<0.001
Yes	604 (49.23)	394 (41.26)	210 (77.21)	

Median (IQR) for continuous variables and counts (percentage) for categorical variables. SAH, subarachnoid hemorrhage; POP, postoperative pneumonia; SpO_2_, percutaneous oxygen saturation; SAPSII, Simplified Acute Physiology Score II; GCS, Glasgow Coma Score; APSIII, Acute Physiology Score III; WFNS, World Federation of Neurosurgical Societies.

**Table 2:** Other clinical data before imputation.

Variable	Overall (*n* = 1,227)	Non-POP (*n* = 955)	POP (*n* = 272)	*p*-value
RDW, %	13.50 (12.90, 14.60)	13.40 (12.90, 14.50)	13.80 (13.20, 15.00)	<0.001
Hemoglobin, g/dL	12.00 (10.60, 13.30)	12.20 (10.70, 13.40)	11.60 (10.20, 12.90)	<0.001
Glucose, mg/dL	129.00 (108.00, 158.00)	127.00 (106.00, 155.00)	135.00 (113.00,172.00)	0.002
WBC, K/μL	10.80 (8.30, 13.80)	10.50 (8.00,13.60)	11.60 (9.10, 14.30)	<0.001
Platelets, K/μL	196.00 (155.00, 245.00)	197.00 (157.00, 248.00)	191.00 (148.00, 238.00)	0.235
Creatinine, mg/dL	0.80 (0.60, 1.00)	0.80 (0.60, 1.00)	0.80 (0.70, 1.10)	0.163
AG, mEq/L	14.00 (12.00, 16.00)	14.00 (12.00, 16.00)	14.00 (12.00, 16.00)	0.489
INR Min	1.10 (1.00, 1.20)	1.10 (1.00, 1.20)	1.10 (1.00, 1.20)	0.561
PT Min	12.10 (11.20, 13.00)	12.10 (11.20, 13.00)	12.00 (11.20, 13.20)	0.785
PTT Min	26.10 (23.90, 28.80)	26.10 (23.90, 28.80)	25.90 (24.00, 28.90)	0.677
SBP, mmHg	127.00 (114.00, 141.00)	128.00 (14.00, 142.00)	126.00 (112.00, 138.00)	0.088
DBP, mmHg	61.00 (54.00,71.00)	62.00 (54.00, 71.00)	61.00 (54.00, 71.00)	0.364

POP, postoperative pneumonia; RDW, red cell distribution width; WBC, white blood cell; AG, anion gap; INR, international normalized ratio; PT, prothrombin time; PTT, partial thromboplastin time; SBP, systolic blood pressure; DBP, diastolic blood pressure.

**Table 3:** Other clinical data after imputation.

Variable	Overall (*n* = 1,227)	Non-POP (*n* = 955)	POP (*n* = 272)	*p*-value
RDW, %	13.50 (12.90, 14.60)	13.50 (12.90,14.40)	13.80 (13.20,14.90)	<0.001
Hemoglobin, g/dL	12.10 (10.60, 13.30)	12.20 (10.70,13.40)	11.60 (10.20,12.90)	<0.001
Glucose, mg/dL	129.00 (108.00, 158.00)	127.00 (107.00,156.00)	135.00 (113.00,172.00)	0.003
WBC, K/μL	10.90 (8.40, 13.70)	10.6 (8.20, 13.50)	11.50 (9.20, 14.20)	<0.001
Platelets, K/μL	197.00 (158.00, 242.00)	197.30 (160.00, 242.00)	194.00 (150.00, 237.00)	0.289
Creatinine, mg/dL	0.80 (0.70, 1.00)	0.80 (0.60, 1.00)	0.80 (0.70, 1.10)	0.248
AG, mEq/L	14.00 (12.20, 16.00)	14.00 (12.20, 15.60)	14.00 (12.70, 16.00)	0.226
INR Min	1.10 (1.00, 1.20)	1.10 (1.00, 1.20)	1.10 (1.00, 1.20)	0.57
PT Min	12.10 (11.20, 13.00)	12.10 (11.20, 12.90)	12.10 (11.20, 13.10)	0.842
PTT Min	26.10 (24.00, 28.70)	26.10 (23.90, 28.70)	26.10 (24.30, 28.60)	0.783
SBP, mmHg	128.00 (121.00, 136.00)	128.00 (122.10, 136.00)	127.00 (115.00, 135.40)	0.099
DBP, mmHg	61.30 (57.00, 66.00)	61.60 (58.00, 66.00)	61.00 (55.00, 68.00)	0.144

POP, postoperative pneumonia; RDW, red cell distribution width; WBC, white blood cell; AG, anion gap; INR, international normalized ratio; PT, prothrombin time; PTT, partial thromboplastin time; SBP, systolic blood pressure; DBP, diastolic blood pressure.

### 3.2 Comparison of the efficacy of different anesthetics in predicting POP in patients with non-traumatic SAH

**Figure 2:**
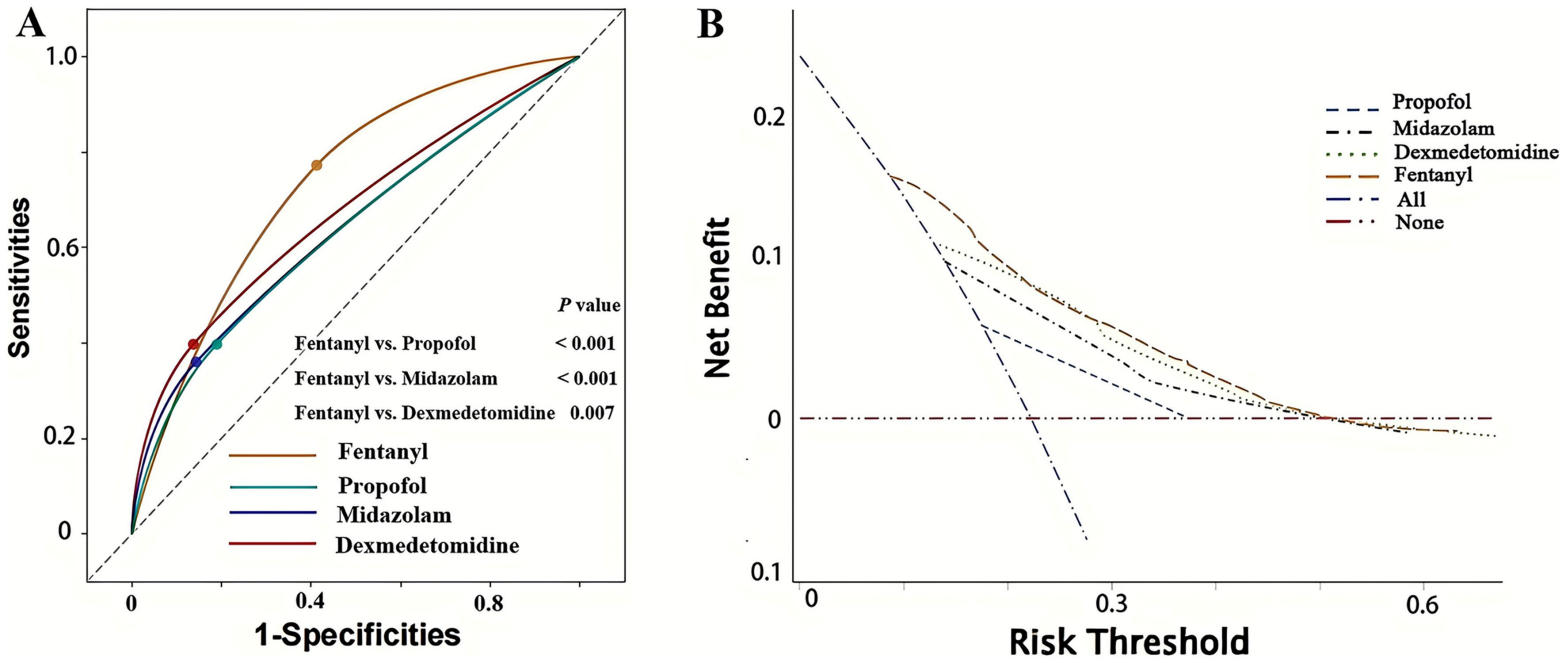
Receiver operating characteristic (ROC) curve analysis and decision curve analysis of four anesthetics in predicting POP in patients with non-traumatic SAH. **(A)** ROC curve analysis; **(B)** decision curve analysis. POP, postoperative pneumonia; SAH, subarachnoid hemorrhage.

**Figure 3:**
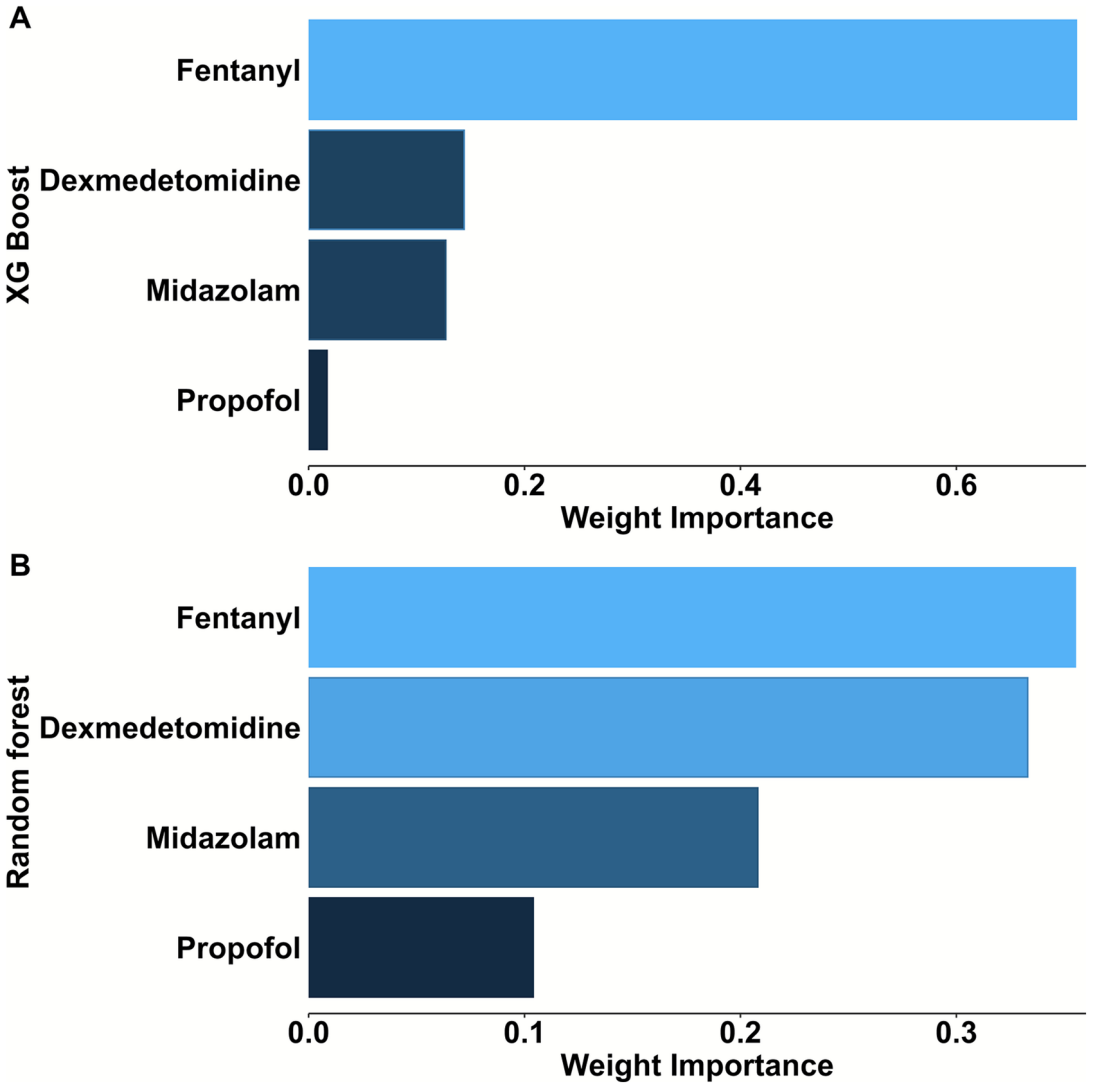
Factors importance analysis of four anesthetics in predicting POP in patients with non-traumatic SAH (ranked from most to least important). **(A)** Random forest method was used in the analysis. **(B)** XGBoost method was used in the analysis. POP, postoperative pneumonia; SAH, subarachnoid hemorrhage; XGBoost, Extreme Gradient Boosting.

To compare the efficacy of different anesthetics in predicting POP, we first conducted ROC analysis and DCA. As shown in Figure 2A, fentanyl (AUC: 0.680 [0.656–0.704]) significantly outperformed propofol (AUC: 0.604 [0.572–0.633]), midazolam (AUC: 0.608 [0.582–0.643]) and dexmedetomidine (AUC: 0.630 [0.601–0.659]) (Delong test: fentanyl vs. propofol: *p* < 0.001; fentanyl vs. midazolam: *p* < 0.001; fentanyl vs. dexmedetomidine: *p* = 0.007) in predicting POP after non-traumatic SAH, while there was no statistically significant difference in AUC among the latter three (all *p* > 0.05). Additionally, the DCA results further confirmed the clinical value of fentanyl in predicting POP (Figure 2B). Besides, fentanyl ranked first for predicting POP, regardless of whether the Random Forest method (Figure 3A) or the XG boost (Extreme Gradient Boosting) method (Figure 3B) was applied. Therefore, subsequent analyses focused on the use of fentanyl as the main exposure factor.

### 3.3 Associations between fentanyl and POP in non-traumatic SAH patients

Multivariate logistic regression models were established to investigate the association between fentanyl and the risk of POP. The multicollinearity analysis results indicate that the VIF for all variables included in the regression models are below 5, suggesting an acceptable level of multicollinearity (Supplementary Table 1). We found that the use of fentanyl was significantly associated with POP in non-traumatic SAH patients after adjusting for various covariates (Table 4). Specifically, in Model 1, which adjusted for demographic characteristics and comorbidities, patients with fentanyl treatment had a 4.979-fold (95%CI: 3.652–6.874) higher odds of developing POP compared to those without fentanyl treatment. In Model 2, after adjustment for vital signs, mechanical ventilation, and traditional scoring systems, the OR decreased to 2.965 (95%CI: 2.138–4.152). Finally, in Model 3, which included laboratory indicators, the OR increased to 4.433 (95%CI: 3.239–6.140). The E-values in Model 1, Model 2, and Model 3 were 9.430, 5.379 and 8.334, respectively. The large E-values indicate that the association between fentanyl and POP is less affected by unmeasured confounding factors.

**Table 4:** Multivariate logistic regression analysis of the association between fentanyl and POP in patients with non-traumatic SAH.

Model 1		Model 2		Model 3	
Variable	OR (95%CI), *p*-value	Variable	OR (95%CI), *p*-value	Variable	OR (95%CI), *p*-value
Fentanyl	4.979 (3.652, 6.874), <0.001	Fentanyl	2.965 (2.138, 4.152), <0.001	Fentanyl	4.433 (3.239, 6.140), <0.001
Age	1.009 (1.000, 1.018), 0.045	Heart rate	1.015 (1.003, 1.026), 0011	RDW	1.085 (0.993, 1.183), 0.068
Gender	1.327 (0.996, 1.771), 0.054	SAPSII	0.992 (0.974, 1.010), 0.398	Hemoglobin	0.917 (0.847, 0.991), 0.030
Congestive heart failure	1.421 (0.903, 2.209), 0.123	APSIII	1.004 (0.993, 1.016), 0.442	Glucose	1.001 (0.999, 1.004), 0.195
Chronic pulmonary disease	1.722 (1.159, 2.538), 0.006	GCS	0.856 (0.821, 0.891), <0.001	WBC	1.021 (0.993, 1.050), 0.139
		Mechanical ventilation	5.384 (2.346, 15.590), <0.001	SPO_2_	0.993 (0.986, 0.999), 0.036

POP, postoperative pneumonia; SAH, subarachnoid hemorrhage; OR, odds ratio; CI, confidence interval; SAPSII, Simplified Acute Physiology Score II; GCS, Glasgow Coma Score; APSIII, Acute Physiology Score III; RDW, red cell distribution width; WBC, white blood cell; SpO_2_, percutaneous oxygen saturation.

### 3.4 Subgroup analyses

**Figure 4:**
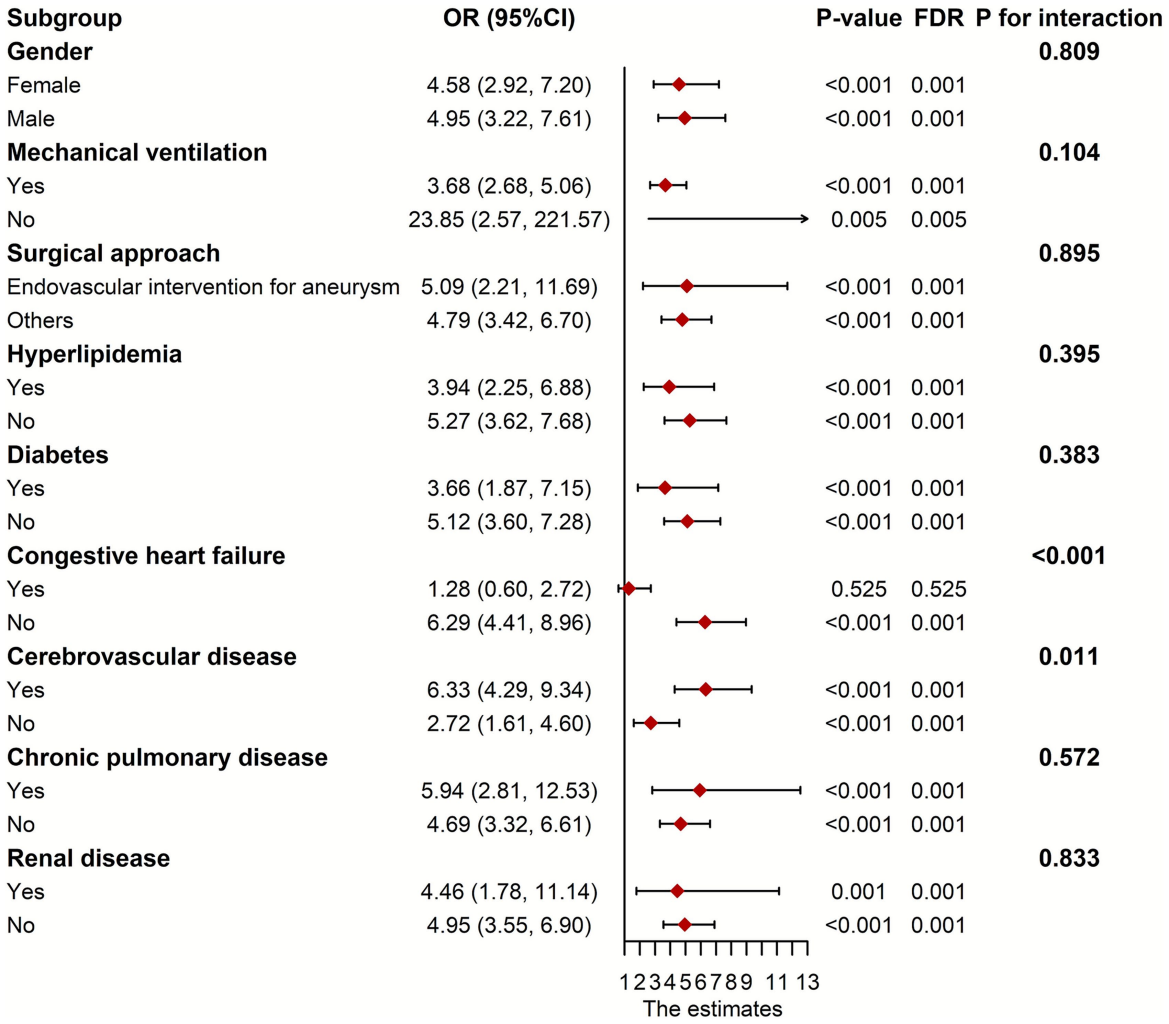
Subgroup analyses between fentanyl and POP. OR, odds ratio; CI, confidence interval; POP, postoperative pneumonia.

**Figure 5:**
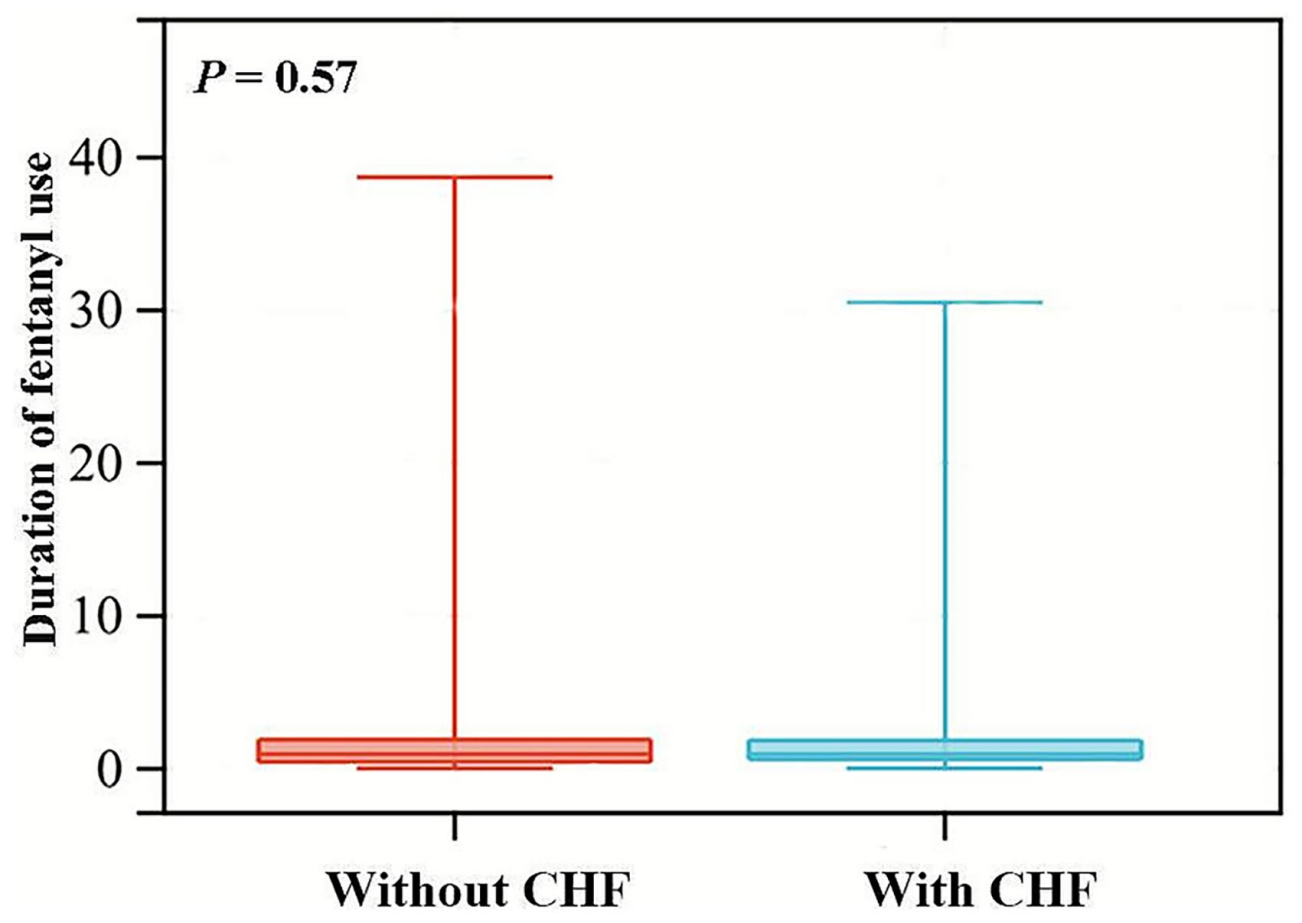
Violin plots for the distribution of fentanyl usage duration among patients with and without CHF. CHF, congestive heart failure.

We stratified the patients according to gender, ventilation status, surgical approach, and preexisting diseases for subgroup analysis. As depicted in Figure 4, CHF and CVD significantly modified the association of fentanyl with POP (*p* for interaction <0.05). In the CHF group, fentanyl was not significantly associated with the risk of POP (OR = 1.278, *p* = 0.525), while in the non-CHF group, fentanyl was significantly associated with the risk of POP (OR = 6.285, *p* < 0.001). This might be caused by the differences in the dosage and duration of fentanyl use, as well as the insufficient sample size of the subgroups. Due to the multiple range intervals of fentanyl dosage in the MIMIC database, it is difficult to classify. Therefore, we only compared the duration of fentanyl use between the two groups, and the results showed no statistically significant difference (Figure 5). The G*Power software was employed to conduct a post-hoc power analysis on the CHF subgroup. The calculated statistical power was 0.699, which falls below the conventional threshold of 0.8. Therefore, the findings derived from this subgroup should be interpreted with caution. In addition, the relationship between fentanyl and POP was significant in both subgroups with or without CVD, but the effect size differed. In patients with CVD, the OR was higher than in those without CVD. Besides, we did not find statistically significant interactions between fentanyl and gender, mechanical ventilation, surgical approach, hyperlipidemia, diabetes, chronic pulmonary disease, and renal disease on POP risk (all *p* for interaction > 0.05). Notably, the significant and non-significant associations remained consistent following FDR correction.

### 3.5 Comparison of the prognostic utility of fentanyl and traditional disease severity scoring systems for predicting POP after non-traumatic SAH

**Figure 6:**
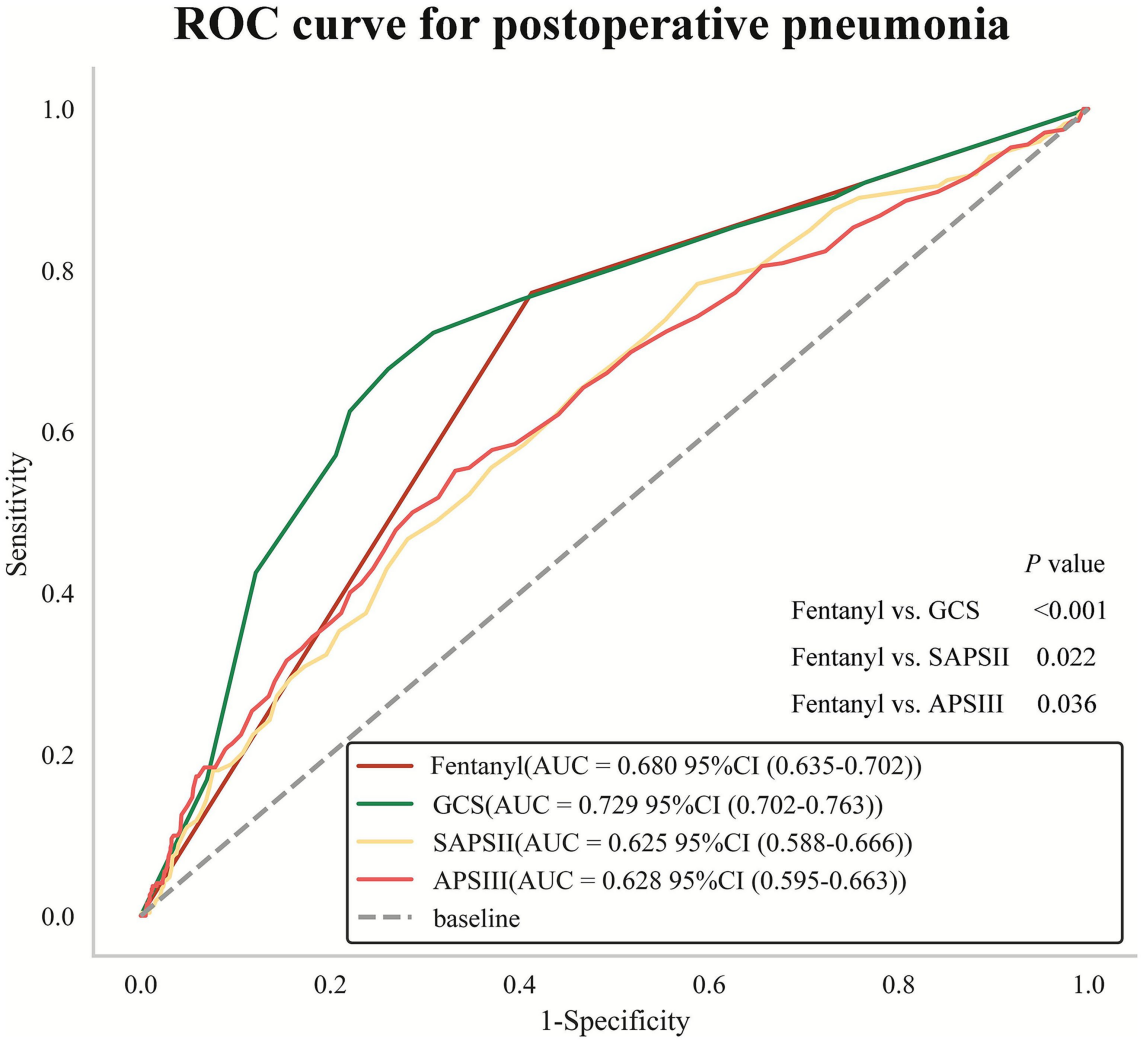
Receiver operating characteristic (ROC) curve analysis for predicting POP. Delong test for comparison of AUC value between fentanyl and GCS, SAPSII, APSIII. POP, postoperative pneumonia; AUC, area under curve; SAPSII, Simplified Acute Physiology Score II; GCS, Glasgow Coma Score; APSIII, Acute Physiology Score III.

The prognosis of patients in the ICU with brain diseases is usually predicted by three disease severity scores: GCS, SAPSII and APSIII (21). Therefore, comparing the predictive efficacy of fentanyl with these scores can evaluate the clinical application value of fentanyl as a predictor of POP in patients with non-traumatic SAH. Further ROC curve analysis and Delong test were conducted to explore the efficacy of fentanyl in predicting POP compared with GCS, SAPSII and APSIII. The AUC values were as follows: fentanyl (AUC = 0.680, 95% CI: 0.635–0.702), GCS (AUC = 0.729, 95% CI: 0.702–0.763), SAPSII (AUC = 0.625, 95% CI: 0.588–0.666), and APSIII (AUC = 0.628, 95% CI: 0.595–0.663). Moreover, the results of Delong tests indicated that the predictive efficacy of fentanyl was significantly superior to SAPSII (*p* = 0.022) and APSIII (*p* = 0.036), yet inferior to GCS (*p* < 0.001) (Figure 6). In IDI analysis, compared with the traditional scoring models, the new models incorporating the fentanyl variable achieved improvements of 4.7% (GCS), 7.0% (SAPSII), and 7.1% (APSIII) in predictive capacity, respectively (Table 5).

**Table 5:** Comparison of changes in predictive efficacy of traditional scoring models with the addition of fentanyl through IDI.

Model	IDI (95%CI)	*p*-value
GCS vs. GCS + Fentanyl	0.047 (0.035, 0.060)	<0.001
SAPSII vs. SAPSII + Fentanyl	0.070 (0.056, 0.085)	<0.001
APSIII vs. APSIII + Fentanyl	0.071 (0.057, 0.086)	<0.001

IDI, integrated discrimination improvement; CI, confidence interval; SAPSII, Simplified Acute Physiology Score II; GCS, Glasgow Coma Score; APSIII, Acute Physiology Score III.

## 4 Discussion

The main finding is that fentanyl demonstrated a higher predictive value for POP than the other anesthetic agents and was independently associated with an increased risk of POP in patients with non-traumatic SAH, even after adjustment for potential confounders. Furthermore, patient comorbidities may modify the relationship between fentanyl and POP. In terms of predictive utility, fentanyl exposure outperformed traditional severity scores such as SAPSII and APSIII, though it was less predictive than the GCS. Most importantly, integrating fentanyl into predictive models significantly enhanced their ability to identify patients at high risk for POP compared to using traditional scores alone.

This study observed that fentanyl was not only significantly associated with POP in patients with non-traumatic SAH but also a more potent predictor of POP than the other three anesthetics. The following mechanisms can explain the association between fentanyl and POP: First, fentanyl has pro-inflammatory effect. Some research has found that long-term or high-dose use of fentanyl may promote the release of pro-inflammatory cytokines (such as IL-6 and TNF-*α*) (22, 23). Second, fentanyl causes immunosuppression. Studies have shown that fentanyl may increase the risk of infection by inhibiting the function of immune cells (such as macrophages, natural killer cells and T-cells), which in turn leads to POP (24). Third, one hypothesized mechanism is that fentanyl may induce coughing. Severe fentanyl-induced cough has been proposed, in isolated reports, to potentially lead to vomiting and aspiration pneumonia (25). Fourth, fentanyl has potential drug–drug interactions. Fentanyl often interacts with other drugs, which may weaken the effect of anti-inflammatory drugs and lead to POP (26). Similarly, propofol and midazolam also have immunosuppressive properties (13), but they have anti-inflammatory effects (27–32). However, although dexmedetomidine lacks immunosuppressive properties (13) and has anti-inflammatory effects (33–36), relevant animal studies have shown that dexmedetomidine does not improve stroke-related pneumonia (16). The above mechanisms may explain the superiority of fentanyl in predicting POP in patients with non-traumatic SAH.

Subgroup analyses revealed that CHF and CVD significantly modified the association of fentanyl with POP. In the CHF group, fentanyl was not significantly associated with the incidence of POP, whereas in the non-CHF group, a significant association was observed. Hormuzdiyar et al. observed CHF was associated with pneumonia after aneurysmal subarachnoid hemorrhage (37), which was consistent with the result of this study. In patients with CHF, varying degrees of alveolar congestion may interfere with the normal physiological functions of alveolar surface fluid at the air-liquid interface and in the lung tissue (including the effective action of surfactant and macrophages), thereby not only increasing the risk of infection but also possibly hindering the clearance of pathogens after infection occurs (38). Therefore, this high-inflammatory microenvironment may weaken the accuracy of fentanyl in predicting POP. We also observed that in patients with CVD, the association was stronger than in those without CVD. One important reason is that patients with combined CVD may have swallowing dysfunction, which makes them more prone to aspiration pneumonia during the perioperative period (39). Secondly, due to the limited range of motion, CVD patients have insufficient pulmonary function exercise, increasing their susceptibility to pulmonary infection (39). Additionally, surgical anesthesia reduces the tension of respiratory muscles, and the anesthetic effect may persist into the postoperative stage (39). Compared with non-CVD patients, CVD patients may have weaker abilities to regulate respiratory muscle function, including the diaphragm (39).

This finding offered a novel perspective for selecting biomarkers in clinical practice, especially when traditional physiological scoring systems exhibit limitations. Unlike SAPSII and APSIII, which primarily focus on systemic physiological dysfunction, fentanyl served as an intervenable drug exposure variable that directly captured iatrogenic risks, thereby enhancing its clinical operability. While the GCS may exhibit a slight advantage over fentanyl due to the inclusion of additional neuro-specific indicators, fentanyl still demonstrated significant independent predictive value. The results of the IDI analysis further confirmed that integrating fentanyl into the existing risk assessment framework not only improved the identification of high-risk patients but also offered more refined guidance for personalized sedation strategies.

The findings of this study hold significant implications for daily clinical practice by addressing a critical gap in the perioperative care of non-traumatic SAH patients. POP is a common and devastating complication, yet traditional risk prediction models often lack anesthesia-specific data. Our analysis provides robust evidence that intraoperative fentanyl exposure is a readily identifiable and independent clinical marker for increased POP risk. This insight allows clinicians to proactively identify a vulnerable patient subgroup as early as the conclusion of surgery. For these high-risk individuals, particularly those with pre-existing CVD or without a history of CHF, our data suggest that clinicians should consider prioritizing alternative non-opioid or non-fentanyl-based analgesic regimens when clinically feasible. More importantly, if fentanyl is deemed necessary, its identification as a risk factor should trigger enhanced postoperative vigilance. This includes implementing structured pulmonary hygiene protocols, earlier mobilization, and closer monitoring for early signs of infection, potentially leading to earlier intervention and improved outcomes.

This study had several limitations. The study employed a retrospective design, which allows for the establishment of an association rather than a causal relationship. Furthermore, although the study involved a substantial number of participating patients, its generalizability may be limited due to the single database source. Future studies conducted among independent populations are needed to verify the results of this research. Since the anesthetic dosage is adjusted according to target-controlled infusion and the target concentration of each drug is different, it is difficult to rule out the influence brought by this situation. Therefore, a major limitation of this study is that we defined anesthetic exposure as a binary variable (yes/no) without quantifying the dose, cumulative exposure. Although this simplification is often necessitated by data availability in large database studies, it could introduce significant confounding bias. For instance, patients who received a small single dose were categorized in the same exposure group as those who received large doses over prolonged infusions. The latter group likely had longer surgery times and more severe underlying conditions, which are intrinsic risk factors for POP. Consequently, the associations we observed, particularly for drugs like fentanyl, might be partially confounded by unmeasured or residual factors such as surgical complexity and illness severity. A critical direction for future prospective research is to perform a more refined quantification of anesthetic exposure (e.g., mg/kg, area under the concentration-time curve) to better elucidate the true relationship between these agents and postoperative outcomes. Moreover, our study is susceptible to residual confounding, particularly confounding by indication. This is a inherent limitation of observational studies in pharmacoepidemiology. Patients who received fentanyl were likely to be different from those who did not; specifically, they may have been more critically ill, had more pain, or undergone different procedures–all factors that could independently influence the risk of developing pneumonia. Although we adjusted for a comprehensive set of covariates, including severity scores (SAPSII, APSIII, GCS), and mechanical ventilation, unmeasured confounding factors (e.g., detailed indications for fentanyl use, physician’s prescribing preference, underlying frailty) may persist. Therefore, the association we observed should not be interpreted as purely causal but rather as a strong signal that warrants further investigation through more robust study designs, such as propensity score-matched analyses or target trial emulation, to better account for this potential bias. In addition, the premature discharge of patients due to severe illness or financial constraints poses a significant challenge in monitoring the occurrence of POP, thereby complicating the assessment of the likelihood of these patients developing POP. The MIMIC-IV database only records the presence or absence of POP during hospitalization, which may limit its applicability in studying the long-term effects of POP. Further long-term prospective research into fentanyl’s efficacy of predicting such postoperative complications is warranted. Finally, this study did not take into account the situation of combined medication, which might have overestimated the effect of fentanyl.

## 5 Conclusion

This study demonstrated that fentanyl was associated with POP in patients with non-traumatic SAH, and this association may be modified by patient comorbidities. In summary, fentanyl use may serve as a predictor for POP in this population, aiding in the early identification of high-risk patients and informing perioperative analgesic management.

## Data Availability

The raw data supporting the conclusions of this article will be made available by the authors, without undue reservation.
