# Supplementary material for: Association between anesthetics and the postoperative pneumonia risk in patients with non-traumatic subarachnoid hemorrhage: an analysis of the MIMIC-IV database
Source: Front Neurol. 2026 Jan 8;16:1615897. doi: 10.3389/fneur.2025.1615897 (PMC12823486; doi:10.3389/fneur.2025.1615897)
Supplement: Supplementary file 1 [file Table_1.DOCX]

**Table 2** Other clinical data before imputation.

| Variable | Overall  (n = 1227) | Non-POP  (n = 955) | POP  (n = 272) | *P*-value |
| --- | --- | --- | --- | --- |
| RDW, % | 13.50 (12.90, 14.60) | 13.40 (12.90, 14.50) | 13.80 (13.20, 15.00) | <0.001 |
| Hemoglobin, g/dL | 12.00 (10.60, 13.30) | 12.20 (10.70, 13.40) | 11.60 (10.20, 12.90) | <0.001 |
| Glucose, mg/dL | 129.00 (108.00, 158.00) | 127.00 (106.00, 155.00) | 135.00 (113.00,172.00) | 0.002 |
| WBC, K/μL | 10.80 (8.30, 13.80) | 10.50 (8.00,13.60) | 11.60 (9.10, 14.30) | <0.001 |
| Platelets, K/μL | 196.00 (155.00, 245.00) | 197.00 (157.00, 248.00) | 191.00 (148.00, 238.00) | 0.235 |
| Creatinine, mg/dL | 0.80 (0.60, 1.00) | 0.80 (0.60, 1.00) | 0.80 (0.70, 1.10) | 0.163 |
| AG, mEq/L | 14.00 (12.00, 16.00) | 14.00 (12.00, 16.00) | 14.00 (12.00, 16.00) | 0.489 |
| INR Min | 1.10 (1.00, 1.20) | 1.10 (1.00, 1.20) | 1.10 (1.00, 1.20) | 0.561 |
| PT Min, s | 12.10 (11.20, 13.00) | 12.10 (11.20, 13.00) | 12.00 (11.20, 13.20) | 0.785 |
| PTT Min, s | 26.10 (23.90, 28.80) | 26.10 (23.90, 28.80) | 25.90 (24.00, 28.90) | 0.677 |
| SBP, mmHg | 127.00 (114.00, 141.00) | 128.00 (14.00, 142.00) | 126.00 (112.00, 138.00) | 0.088 |
| DBP, mmHg | 61.00 (54.00,71.00) | 62.00 (54.00, 71.00) | 61.00 (54.00, 71.00) | 0.364 |

Abbreviations: POP, postoperative pneumonia; RDW, red cell distribution width; WBC, white blood cell; AG, anion gap; INR, international normalized ratio; PT, prothrombin time; PTT, partial thromboplastin time; SBP, systolic blood pressure; DBP, diastolic blood pressure.
