# Supplementary material for: Association between anesthetics and the postoperative pneumonia risk in patients with non-traumatic subarachnoid hemorrhage: an analysis of the MIMIC-IV database
Source: Front Neurol. 2026 Jan 8;16:1615897. doi: 10.3389/fneur.2025.1615897 (PMC12823486; doi:10.3389/fneur.2025.1615897)
Supplement: Supplementary file 2 [file Table_2.DOCX]

**Table 3** Other clinical data after imputation.

| Variable | Overall  (n = 1227) | Non-POP  (n = 955) | POP  (n = 272) | *P*-value |
| --- | --- | --- | --- | --- |
| RDW, % | 13.50 (12.90, 14.60) | 13.50 (12.90,14.40) | 13.80 (13.20,14.90) | <0.001 |
| Hemoglobin, g/dL | 12.10 (10.60, 13.30) | 12.20 (10.70,13.40) | 11.60 (10.20,12.90) | <0.001 |
| Glucose, mg/dL | 129.00 (108.00, 158.00) | 127.00 (107.00,156.00) | 135.00 (113.00,172.00) | 0.003 |
| WBC, K/μL | 10.90 (8.40, 13.70) | 10.6 (8.20, 13.50) | 11.50 (9.20, 14.20) | <0.001 |
| Platelets, K/μL | 197.00 (158.00, 242.00) | 197.30 (160.00, 242.00) | 194.00 (150.00, 237.00) | 0.289 |
| Creatinine, mg/dL | 0.80 (0.70, 1.00) | 0.80 (0.60, 1.00) | 0.80 (0.70, 1.10) | 0.248 |
| AG, mEq/L | 14.00 (12.20, 16.00) | 14.00 (12.20, 15.60) | 14.00 (12.70, 16.00) | 0.226 |
| INR Min | 1.10 (1.00, 1.20) | 1.10 (1.00, 1.20) | 1.10 (1.00, 1.20) | 0.57 |
| PT Min, s | 12.10 (11.20, 13.00) | 12.10 (11.20, 12.90) | 12.10 (11.20, 13.10) | 0.842 |
| PTT Min, s | 26.10 (24.00, 28.70) | 26.10 (23.90, 28.70) | 26.10 (24.30, 28.60) | 0.783 |
| SBP, mmHg | 128.00 (121.00, 136.00) | 128.00 (122.10, 136.00) | 127.00 (115.00, 135.40) | 0.099 |
| DBP, mmHg | 61.30 (57.00, 66.00) | 61.60 (58.00, 66.00) | 61.00 (55.00, 68.00) | 0.144 |

Abbreviations: POP, postoperative pneumonia; RDW, red cell distribution width; WBC, white blood cell; AG, anion gap; INR, international normalized ratio; PT, prothrombin time; PTT, partial thromboplastin time; SBP, systolic blood pressure; DBP, diastolic blood pressure.
