# Supplementary material for: Association between anesthetics and the postoperative pneumonia risk in patients with non-traumatic subarachnoid hemorrhage: an analysis of the MIMIC-IV database
Source: Front Neurol. 2026 Jan 8;16:1615897. doi: 10.3389/fneur.2025.1615897 (PMC12823486; doi:10.3389/fneur.2025.1615897)
Supplement: Supplementary file 3 [file Table_3.DOCX]

**Table 4** Multivariate logistic regression analysis of the association between fentanyl and POP in patients with non-traumatic SAH

| Model 1 | | Model 2 | | Model 3 | |
| --- | --- | --- | --- | --- | --- |
| Variable | OR (95%CI), *P*-value | Variable | OR (95%CI), *P*-value | Variable | OR (95%CI), *P*-value |
| Fentanyl | 4.979 (3.652, 6.874), <0.001 | Fentanyl | 2.965 (2.138, 4.152), <0.001 | Fentanyl | 4.433 (3.239, 6.140), <0.001 |
| Age | 1.009 (1.000, 1.018), 0.045 | Heart rate | 1.015 (1.003, 1.026), 0011 | RDW | 1.085 (0.993, 1.183), 0.068 |
| Gender | 1.327 (0.996, 1.771), 0.054 | SAPSII | 0.992 (0.974, 1.010), 0.398 | Hemoglobin | 0.917 (0.847, 0.991), 0.030 |
| Congestive heart failure | 1.421 (0.903, 2.209), 0.123 | APSIII | 1.004 (0.993, 1.016), 0.442 | Glucose | 1.001 (0.999, 1.004), 0.195 |
| Chronic pulmonary disease | 1.722 (1.159, 2.538), 0.006 | GCS | 0.856 (0.821, 0.891), <0.001 | WBC | 1.021 (0.993, 1.050), 0.139 |
|  |  | Mechanical ventilation | 5.384 (2.346, 15.590), <0.001 | SPO_2_ | 0.993 (0.986, 0.999), 0.036 |

Abbreviations: POP, postoperative pneumonia; SAH, subarachnoid hemorrhage; OR, odds ratio; CI, confidence interval; SAPSII, Simplified Acute Physiology Score II; GCS, Glasgow Coma Score; APSIII, Acute Physiology Score III; RDW, red cell distribution width; WBC, white blood cell; SpO_2_, percutaneous oxygen saturation.
