# Supplementary material for: Association between anesthetics and the postoperative pneumonia risk in patients with non-traumatic subarachnoid hemorrhage: an analysis of the MIMIC-IV database
Source: Front Neurol. 2026 Jan 8;16:1615897. doi: 10.3389/fneur.2025.1615897 (PMC12823486; doi:10.3389/fneur.2025.1615897)
Supplement: Supplementary file 4 [file Table_4.DOCX]

**Table 5** Comparison of changes in predictive efficacy of traditional scoring models with the addition of fentanyl through IDI

| Model | IDI (95%CI) | *P*-value |
| --- | --- | --- |
| GCS vs. GCS + Fentanyl | 0.047 (0.035, 0.060) | <0.001 |
| SAPSII vs. SAPSII + Fentanyl | 0.070 (0.056, 0.085) | <0.001 |
| APSIII vs. APSIII + Fentanyl | 0.071 (0.057, 0.086) | <0.001 |

Abbreviations: IDI, integrated discrimination improvement; CI, confidence interval; SAPSII, Simplified Acute Physiology Score II; GCS, Glasgow Coma Score; APSIII, Acute Physiology Score III.
