# Supplementary material for: Association between anesthetics and the postoperative pneumonia risk in patients with non-traumatic subarachnoid hemorrhage: an analysis of the MIMIC-IV database
Source: Front Neurol. 2026 Jan 8;16:1615897. doi: 10.3389/fneur.2025.1615897 (PMC12823486; doi:10.3389/fneur.2025.1615897)
Supplement: Supplementary file 5 [file Table_5.DOCX]

**Supplementary Table 1.** Multicollinearity analysis between variables

| Variables | VIF |
| --- | --- |
| Age | 1.558 |
| gender | 1.071 |
| Congestive heart failure | 1.144 |
| Chronic pulmonary disease | 1.054 |
| Heart rate | 1.120 |
| Mechanical ventilation | 1.187 |
| SAPSII | 3.634 |
| APSIII | 3.023 |
| GCS | 1.439 |
| RDW | 1.360 |
| Hemoglobin | 1.375 |
| Glucose | 1.153 |
| WBC | 1.211 |
| SpO_2_ | 1.079 |
| Fentanyl | 1.244 |
